# Salvage thoracoscopic esophagectomy after carbon-ion radiotherapy in a patient with esophageal squamous cell carcinoma: a case report

**DOI:** 10.1186/s40792-022-01372-2

**Published:** 2022-02-03

**Authors:** Kengo Kuriyama, Makoto Sohda, Hideyuki Saito, Yasunari Ubukata, Nobuhiro Nakazawa, Keigo Hara, Makoto Sakai, Akihiko Sano, Hiroomi Ogawa, Takaaki Sano, Shigeo Yasuda, Hitoshi Ishikawa, Ken Shirabe, Hiroshi Saeki

**Affiliations:** 1 Division of Gastroenterological Surgery, Department of General Surgical Science, Graduate School of Medicine, Gunma University, 3-39-22 Showa-machi, Maebashi, Gunma 371-8511 Japan; 2 Department of Diagnostic Pathology, Gunma University Graduate School of Medicine, Maebashi, Japan; 3 National Institutes for Quantum Science and Technology, QST Hospital, Chiba, Japan; 4 Department of General Surgical Science, Gunma University Graduate School of Medicine, Maebashi, Gunma Japan

**Keywords:** Esophageal cancer, Salvage surgery, Thoracoscopic surgery, Carbon-ion radiotherapy

## Abstract

**Background:**

Carbon-ion radiotherapy (CIRT) for esophageal cancer has been receiving significant attention given its high local control rates and minimal damage to normal tissues. However, the efficacy and safety of salvage surgery after CIRT for esophageal cancer remain unclear. We report the case of a patient who underwent salvage thoracoscopic surgery after CIRT.

**Case presentation:**

A 51-year-old woman underwent upper gastrointestinal endoscopy and a type 0-IIa + 0-IIc esophageal squamous cell carcinoma located 27–29 cm from the patient’s incisors, classified as clinical stage I (T1bN0M0), was detected. She received CIRT (50.4 Gy [relative biological effectiveness, RBE]/12 fr) for localized esophageal cancer and achieved complete remission after 4 months. Six years after CIRT, follow-up endoscopic examination demonstrated a type 0-IIa + 0-IIc tumor in the previously treated area. In addition, a type 0-IIa lesion located 20–22 cm from the incisors was found. We diagnosed localized ESCC, classified as clinical stage I (T1bN0M0). Salvage thoracoscopic surgery was performed in the prone position with five access ports. Although the esophagus tightly adhered to the thoracic descending aorta and left main bronchus with severe fibrosis, the esophagus could be separated from the surrounding organs with careful forceps manipulation. The operation time and blood loss were 8 h 45 min and 253 mL, respectively. The patient was discharged from our hospital 17 days after the salvage surgery without any complications. Pathological findings revealed two squamous cell carcinomas. Both tumors were localized in the lamina propria mucosa, and lymph node metastasis was not detected. The tumors were diagnosed as pathological stage IA (pT1aN0M0) according to the TNM criteria. Moreover, pathological examinations showed severe fibrosis of the previously irradiated tissues compared to the normal esophagus located outside of the irradiation field. Following the surgery, the patient had no recurrence for 1 year and 6 months.

**Conclusions:**

Thoracoscopic radical esophagectomy can be performed as salvage surgery. Careful and discreet surgery is integral to perform salvage surgery after CIRT since CIRT may cause severe adhesions and fibrosis in the irradiated field.

## Background

Esophageal cancer is the sixth most common cause of cancer-related deaths worldwide [1]. Esophageal squamous cell carcinoma (ESCC) is the most common histological subtype of esophageal cancer in many countries, including Japan [2, 3]. Definitive chemoradiotherapy (dCRT) represents a treatment option for patients with ESCC. Although many patients have been cured using dCRT, the overall survival rate of patients having received dCRT remains 32.4% [4].

In light of advancements in irradiation machinery, carbon-ion radiotherapy (CIRT) has been increasingly used alongside stereotactic body radiation therapy. CIRT has higher local control rates and incurs minimal damage to normal tissues thanks to a higher local dose distribution than conventional radiotherapy using photons. Several studies have documented the effectiveness of CIRT for esophageal cancer [5, 6]; however, some patients experience remnant tumor or local recurrence. Although salvage surgery may be considered in patients with remnant or recurrent ESCC after CIRT who can tolerate surgery, its efficacy and safety remain unclear.

Here, we document the case of a patient with ESCC who underwent thoracoscopic esophagectomy following CIRT as salvage surgery. To our knowledge, this is the first report of salvage esophagectomy following CIRT.

## Case presentation

**Fig. 1:**
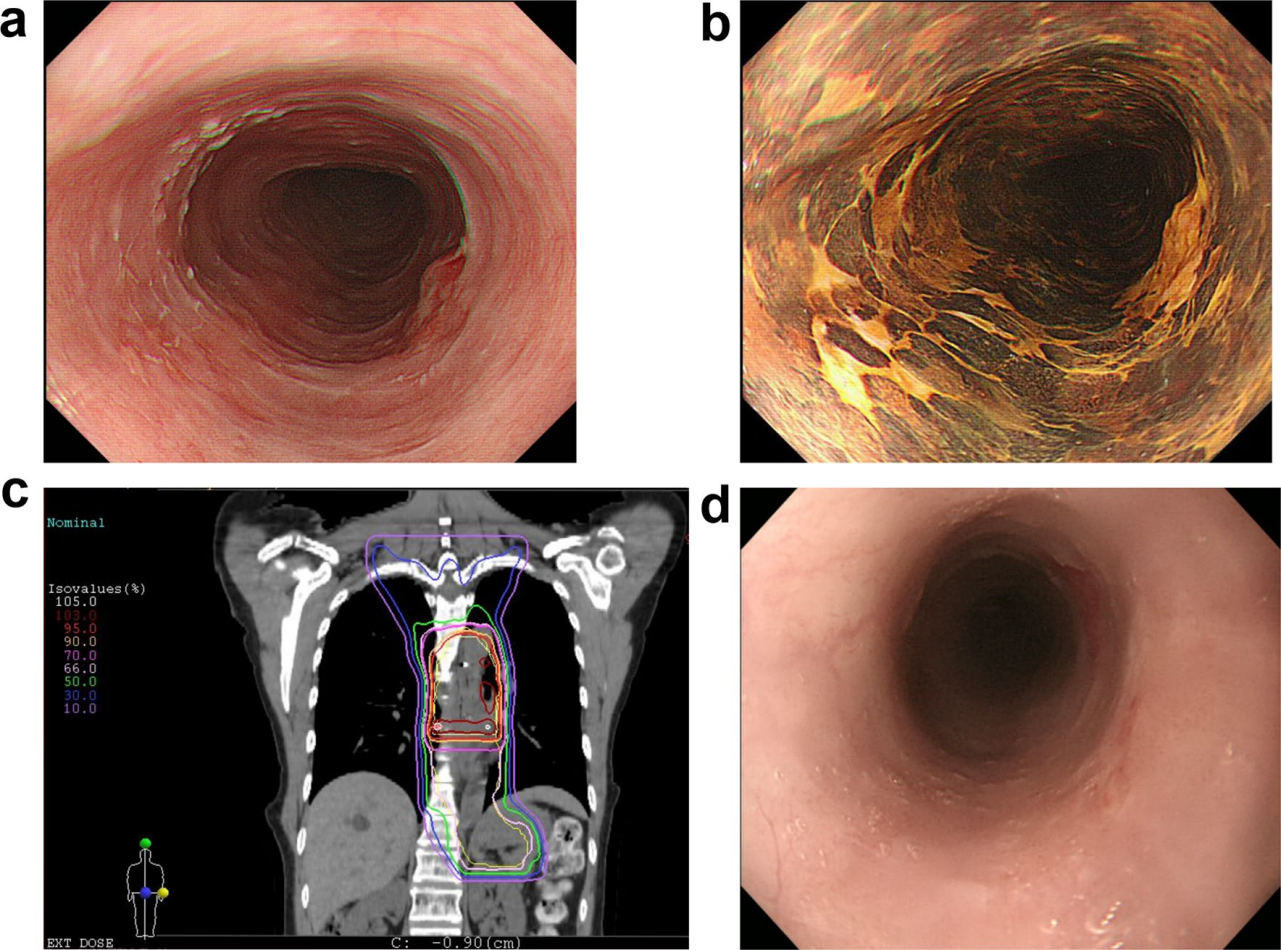
Endoscopic examination prior to and after carbon-ion radiotherapy. **a** Upper gastrointestinal endoscopy showing a type 0-IIa + 0-IIc lesion located 27–29 cm from the incisors. **b** Chromoendoscopy with iodine staining revealing a lugol-voiding lesion with distinct margin. **c** Dose distribution of the carbon-ion radiotherapy. The isodose line of 95% is indicated by the orange line. **d** Examination 4 months after carbon-ion radiotherapy. The patient achieved a complete response

A 51-year-old female underwent upper gastrointestinal endoscopy during a medical checkup. An examination identified a type 0-IIa + 0-IIc lesion located 27–29 cm from the patient’s incisors (Fig. 1a), and chromoendoscopy with iodine staining demonstrated a lugol-voiding lesion with distinct margin (Fig. 1b). A pathological examination of the endoscopic biopsy revealed squamous cell carcinoma. Contrast-enhanced computed tomography (CT) and ^18^F-fluorodeoxyglucose positron emission tomography (FDG-PET) did not detect any lymph nodes or distant metastases. The patient was diagnosed with middle intrathoracic ESCC, classified as clinical stage I (T1bN0M0) according to the UICC-TNM classification 8th edition [7]. Since the patient requested non-surgical treatment, she had undergone CIRT (50.4 Gy [relative biological effectiveness, RBE]/12 fr) without concurrent chemotherapy for localized esophageal cancer at the National Institutes for Quantum Science and Technology, QST Hospital (Fig. 1c). Four months thereafter, the patient achieved complete remission (Fig. 1d).

**Fig. 2:**
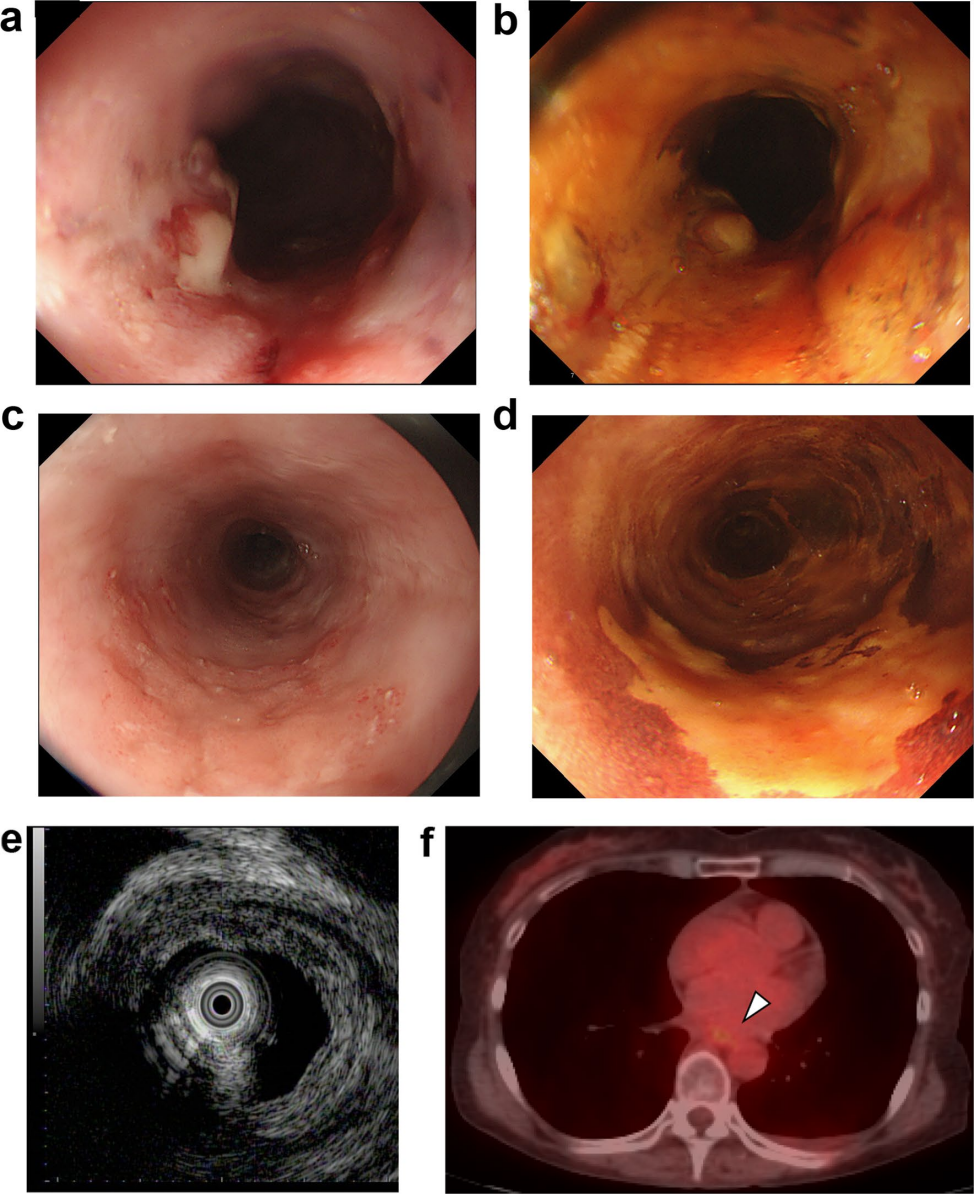
Findings prior to salvage surgery. **a**–**d** Upper gastrointestinal endoscopy 6 years after carbon-ion radiotherapy. An elevated lesion at the previously treated area (27–29 cm from the incisors) was detected by **a** white light endoscopy and **b** Lugol staining. In addition, a type 0-IIa lesion located 20–22 cm from the incisors had freshly appeared. **c** White light endoscopy. **d** Lugol staining. **e** Ultrasound endoscopic finding. The tumor located 27–29 cm from the incisors was thought to have infiltrated the submucosal layer. **f** ^18^F-deoxyglucose (FDG) positron emission tomography revealing slight FDG uptake in the middle esophagus (white arrowhead)

Six years after CIRT, a follow-up endoscopic examination demonstrated a type 0-IIa + 0-IIc tumor in the previously treated area (27–29 cm from the incisors, Fig. 2a, b). In addition, a type 0-IIa lesion located 20–22 cm from the incisors freshly appeared (Fig. 2c, d). A biopsy of both lesions revealed a squamous cell carcinoma on pathological examination. The tumor, located 27–29 cm from the incisors, was thought to have infiltrated the submucosal layer according to the ultrasound endoscopic findings (Fig. 2e). FDG-PET showed slight FDG uptake (SUVmax 2.6) in the middle esophagus (Fig. 2f). Contrast-enhanced computed tomography and fluorodeoxyglucose positron emission tomography revealed no metastatic sites. We diagnosed a localized ESCC, classified as clinical stage I (T1bN0M0). An endoscopic resection was difficult because the tumor was located on the scar. Thus, we suggested either palliative chemotherapy or salvage thoracoscopic surgery to the patient. She chose to undergo salvage thoracoscopic esophagectomy after having processed the associated treatment risks.

**Fig. 3:**
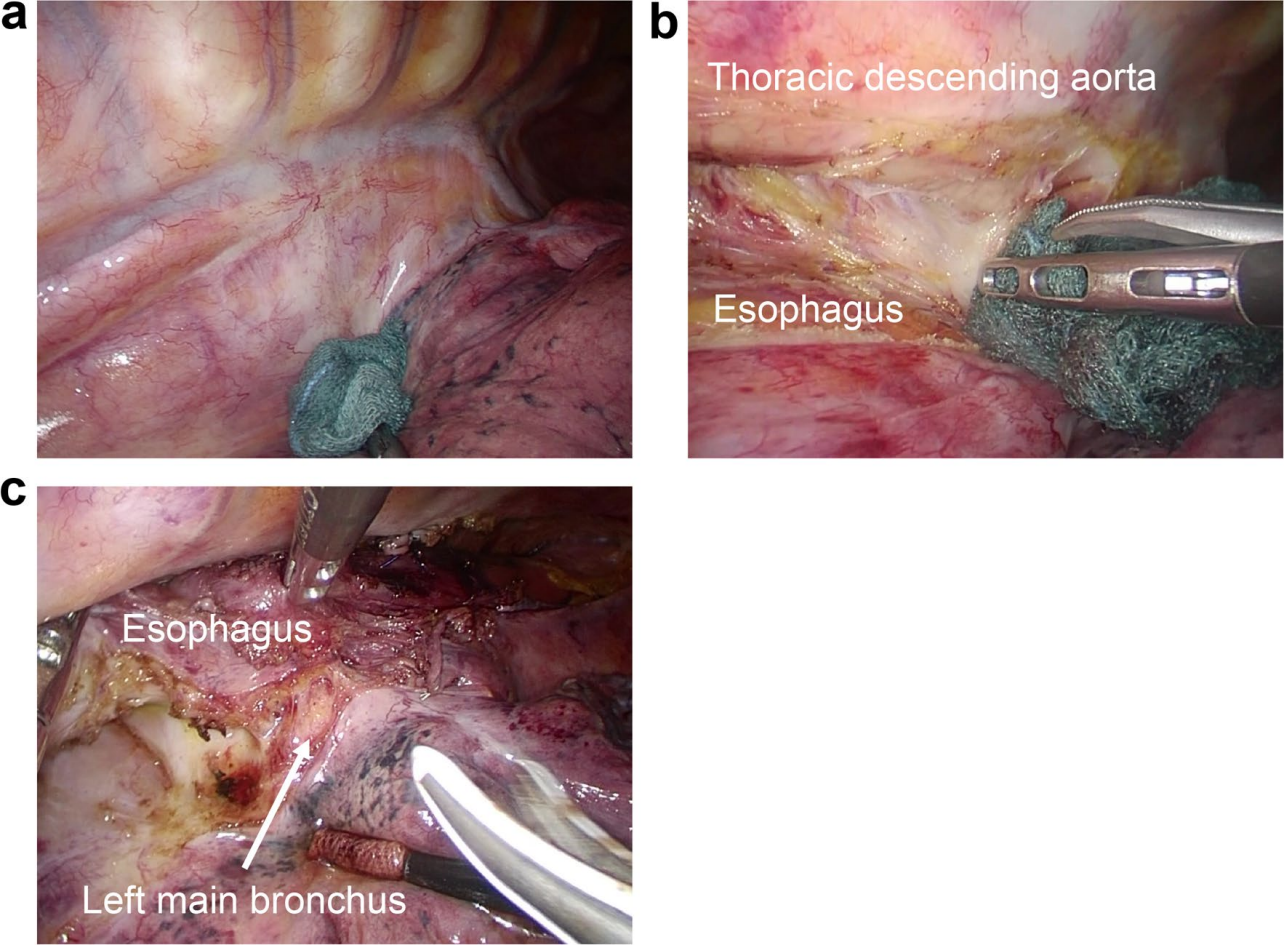
Intraoperative findings. **a** The tumor was located in the middle esophagus. The esophageal adventitia had white scarring. **b**, **c** The esophagus tightly adhered to the thoracic descending aorta and left main bronchus

Salvage thoracoscopic surgery was performed with the patient in a prone position with five access ports. The esophagus was tightly adhered to the thoracic descending aorta and left main bronchus with severe fibrosis (Fig. 3a–c). Although it took a lot of time to dissect the adhesion, the esophagus was able to be successfully separated from the descending aorta and left main bronchus by careful forceps manipulation. Following the thoracoscopic surgery, the patient was placed in a supine position. The gastric tube was created and raised through the retrosternal route using laparoscopic surgical methods. The surgery lasted 8 h and 45 min and a total of 253 mL of blood was lost. No complications occurred during the postoperative period. The patient was discharged from our hospital 17 days after the salvage surgery.

**Fig. 4:**
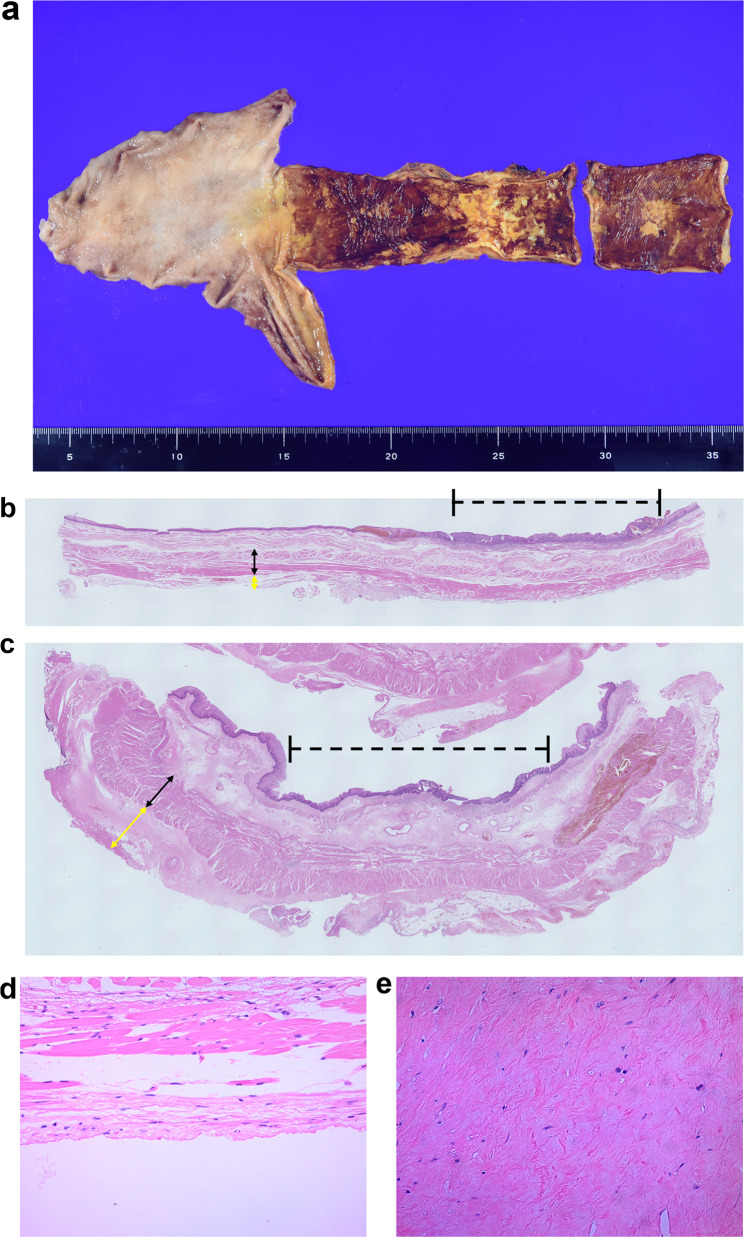
Pathological findings. **a** Macroscopic findings revealed two squamous cell carcinomas. One was located in the upper thoracic esophagus, and the other was located in the middle thoracic esophagus. **b**, **c** Hematoxylin and eosin staining of the tumor located in the **b** unirradiated upper thoracic esophagus and **c** previously irradiated middle thoracic esophagus. The dashed black lines point to the area of each tumor. Black and yellow arrows show the thickness of the muscularis propria and adventitia, respectively. **d**, **e** Microscopic findings of the adventitia of the **d** unirradiated normal esophagus and **e** previously irradiated lesion (original magnification × 200). The adventitia of the irradiated esophagus had severe fibrosis and thickness compared to the normal esophagus located outside the irradiation field

Pathological findings revealed two squamous cell carcinomas. One was located in the upper thoracic esophagus, and the other was located in the middle thoracic esophagus (Fig. 4a). The muscularis propria and adventitia in the middle thoracic esophagus, which had already been irradiated by CIRT 6 years ago, were thicker than those in the upper thoracic esophagus, which had not been previously irradiated (Fig. 4b, c). Both tumors were located in the lamina propria mucosa (LPM), and no lymph node metastasis was detected. Therefore, the tumors were diagnosed as pathological stage IA (pT1aN0M0) according to the TNM criteria. Postoperatively, the patient experienced no recurrence for 1 year and 6 months.

## Discussion

Esophagectomy using thoracoscopy and/or laparoscopy, the so-called minimally invasive esophagectomy (MIE), is increasingly performed worldwide. According to a recent review article, the incidence rates of postoperative complications after MIE, such as pneumonia, arrhythmia, anastomotic leakage, and recurrent laryngeal nerve palsy, are approximately 10% each [8]. Salvage esophagectomy is performed in patients with remaining or recurrent ESCC after dCRT and is associated with high rates of morbidity and mortality. Watanabe et al. reported that postoperative complications occurred in 65.1% of patients; the mortality rate was 7.9% [9]. In recent years, MIE as salvage surgery has also been performed; Takemura et al. performed salvage thoracoscopic esophagectomy following dCRT in 27 patients without serious perioperative complications [10]. In general, thoracoscopic esophagectomy presents a number of advantages, such as magnification effect and good lightning, although it remains technically demanding and associated with a significant operator learning curve [11]. In this case, thoracoscopic esophagectomy was carried out as a salvage treatment after CIRT without any perioperative complications. Salvage MIE after CIRT may be an option for esophageal resection.

Although several studies have demonstrated the treatment efficacy of CIRT for ESCC [5, 6], no case of salvage esophagectomy following CIRT has yet been published. Thus, the impact of CIRT on esophageal surgery remains unclear. In a mouse model, Cui et al. reported that irradiation using carbon ions induced more severe xenograft tumor fibrosis than did X-rays [12]. Ohtaki et al. showed that CIRT resulted in strong fibrosis and adhesion inside the therapeutic dose irradiation field in patients with lung cancer [13]. In their study, five of the six patients with lung cancer required a combined resection, which would not be necessary before CIRT given the presence of strong adhesions and the possibility of tumor extension. According to these previous reports, CIRT may induce more severe fibrosis of irradiated tissues than X-rays. However, it is difficult to declare whether X-rays or CIRT causes stronger fibrosis of esophageal cancer with only our one case. An accumulation of additional cases is needed to determine whether X-rays or CIRT induces more severe fibrosis in patients with esophageal cancer. In our study, we dissected the adhesion between the esophagus and the surrounding organs by thoracoscopic surgery. However, pathological examinations revealed severe fibrosis of the previously irradiated tissues compared to the normal esophagus located outside of the irradiation field (Fig. 4d, e). To minimize damage to other organs, it is necessary to identify which organs should or should not be preserved, and then separate them carefully.

There have been no randomized trials comparing clinical results of X-rays and CIRT. It is difficult to discuss the therapeutic results of CIRT when compared with those of X-rays because there have been few reports on their treatment outcomes and the number of cases treated is small. Enrollment for a phase I/II clinical trial of CIRT for patients with clinical stage I ESCC (UMIN000013552) has already been completed and the results of the analysis are awaited.

Prior to surgery, we found that the tumor had infiltrated the submucosal layer according to the endoscopic ultrasonography (EUS) findings. However, a pathological examination revealed that the tumor was located in the LPM. Typically, it remains difficult to accurately predict the depth of the tumor following irradiation due to tissue fibrosis. Griffin et al. showed that the accuracy rate of EUS for esophageal cancer following neoadjuvant chemoradiotherapy was 36% [14]. Although it remains unknown whether EUS can diagnose the depth of the tumor after CIRT, we believe that EUS after CIRT is also less accurate after chemoradiotherapy. However, an endoscopic resection was difficult to perform because the tumor was located on the scar. We believe that radical esophagectomy as a salvage treatment was indispensable in this case.

In this case, 6 years after CIRT, a new esophageal cancer developed at the site of the primary tumor. It remains unknown whether a newly appearing cancer is a recurrent primary tumor or metachronous disease. Since we did not perform a genomic analysis, it was difficult to correctly answer this oncological question. However, Xi et al. reported that 88.3% of recurrences after chemoradiotherapy in patients with esophageal cancer occurred within 2 years [15]. We speculate that a metachronous tumor freshly appeared 6 years after CIRT in our case. It remains unclear whether CIRT for esophageal cancer increases the risk of subsequent primary cancer. However, CIRT can show high local control rates and achieve long-term survival. Long-term follow-up is desirable after CIRT in patients with esophageal cancer.

## Conclusion

Although CIRT caused strong fibrosis and adhesion between the tumor and surrounding tissues, we performed a thoracoscopic radical esophagectomy as salvage surgery. Careful and discreet surgery is integral to perform salvage surgery after CIRT since CIRT may cause severe adhesions and fibrosis in the irradiated field.

## Abbreviations

ESCC: Esophageal squamous cell carcinoma
dCRT: Definitive chemoradiotherapy
CIRT: Carbon-ion radiotherapy
CT: Computed tomography
FDG-PET: ^18^F-fluorodeoxyglucose positron emission tomography
RBE: Relative biological effectiveness
LPM: Lamina propria mucosa
MIE: Minimally invasive esophagectomy
EUS: Endoscopic ultrasonography
